# Expression of concern: Unprecedented inhibition of glycosidase-catalyzed substrate hydrolysis by nanodiamond-grafted *O*-glycosides

**DOI:** 10.1039/d4ra90137a

**Published:** 2024-12-06

**Authors:** Aloysius Siriwardena, Manakamana Khanal, Alexandre Barras, Omprakash Bande, Teresa Mena-Barragán, Carmen Ortiz Mellet, José Manuel Garcia Fernández, Rabah Boukherroub, Sabine Szunerits

**Affiliations:** a Laboratoire de Glycochimie des Antimicrobiennes et Bioresources, FRE-CNRS 3517, Université de Picardie Jules Verne 80039 Amiens France aloysius.siriwardena@u-picardie.fr; b Institute of Electronics, Microelectronics and Nanotechnology (IEMN), UMR-CNRS 8520, Lille1 University Avenue Poincaré-BP 60069, 59652 Villeneuve d'Ascq France sabine.szunerits@iri.univ-lille1.fr; c Faculty of Chemistry, University of Sevilla C/Profesor Garcia Gonzalez 1 E-41012 Sevilla Spain mellet@us.es; d Instituto de Investigaciones Químicas (IIQ), CSIC – Universidad de Sevilla Avda. Américo Vespucio 49 E-41092 Sevilla Spain jogarcia@iiq.csic.es

## Abstract

Expression of concern for ‘Unprecedented inhibition of glycosidase-catalyzed substrate hydrolysis by nanodiamond-grafted *O*-glycosides’ by Aloysius Siriwardena *et al.*, *RSC Adv.*, 2015, **5**, 100568–100578, https://doi.org/10.1039/C5RA21390H.

The Royal Society of Chemistry is publishing this expression of concern in order to alert readers that concerns have been raised regarding the reliability of the data. The Royal Society of Chemistry has asked the University of Lille to investigate this matter. An expression of concern will continue to be associated with the article until we receive conclusive evidence regarding the reliability of the reported data.

Laura Fisher

5th November 2024

Executive Editor, *RSC Advances*

